# Supplementary material for: Strain-Dependent Variability in Ochratoxin A Production by Aspergillus spp. Under Different In Vitro Cultivation Conditions
Source: Microorganisms. 2025 Dec 15;13(12):2850. doi: 10.3390/microorganisms13122850 (PMC12735859; doi:10.3390/microorganisms13122850)
Supplement: Supplementary file 1 [file microorganisms-13-02850-s001.zip › microorganisms-3934091-supplementary.pdf]

Table S1. General linear model and effect size (η2 a R^2 coefficients) table for OTA concentration measured in 5 timepoints for 9 *Aspergillus* strains cultivated on 4 different media in 4 different temperatures.

Factor	Df	Sum Sq	Mean Sq	F value	Pr (>F)	η2	R^2
Strain	8	3174.68	396.83	2583.2	< 0.0001	0.935	0.438
Medium	3	1151.45	383.82	2498.5	< 0.0001	0.839	0.159
Temperature	3	13.13	4.38	28.5	< 0.0001	0.056	0.002
Day	4	66.81	16.70	108.7	< 0.0001	0.232	0.009
Strain*Medium	24	626.55	26.11	169.9	< 0.0001	0.739	0.086
Strain*Temperature	24	268.64	11.19	72.9	< 0.0001	0.548	0.037
Strain*Day	32	281.04	8.78	57.2	< 0.0001	0.560	0.039
Medium*Temperature	9	70.09	7.79	50.7	< 0.0001	0.241	0.010
Medium*Day	12	32.16	2.68	17.4	< 0.0001	0.127	0.004
Temperature*Day	12	32.48	2.71	17.6	< 0.0001	0.128	0.004
Strain*Medium*Temperature	72	392.58	5.45	35.5	< 0.0001	0.640	0.054
Strain*Medium*Day	96	222.14	2.31	15.1	< 0.0001	0.501	0.031
Strain*Temperature*Day	96	249.95	2.60	16.9	< 0.0001	0.531	0.034
Medium*Temperature*Day	36	63.65	1.77	11.5	< 0.0001	0.223	0.009
Strain*Medium*Temperature*Day	288	387.84	1.35	8.8	< 0.0001	0.637	0.053
Residuals	1440	221.21	0.15	-	-	-	0.030

Notes: Df – degrees of freedom, Sum Sq - sum of square, Mean Sq - mean squares, η2 - eta squared, R^2 - coefficient of determination, Pr (>F) stastical significance

Table S2. Average maximum OTA concentrations (μg g^{-1}) produced by *Aspergillus* strains under different cultivation media and temperatures.

Strain no.	Strain	t (°C)	Medium CYA/day	MEA/day	PDA/day	YES/day	All media
1	*A. ochraceus* IFA/Austria	18	25.69 b/6	3.43 a/6	2.29 a/6	92.36 c/10	30.94 c
		22	21.43 b/6	2.1 a/6	1.71 a/6	27.85 b/21	13.27 b
		25	7.03 a/6	<LOD a/6	1.55 a/6	32.26 b/10	10.21 ab
		30	2.58 a/6	10.15 b/30	5.66 a/6	11.33 a/14	7.43 a
2	*A. ochraceus* Biomin/Austria	18	88.79 c/30	3.87 a/10	17.3 b/30	848.34 b/30	239.57 b
		22	55.28 b/10	<LOD a/10	5.95 a/30	132.39 a/21	48.4 a
		25	123.07 d/14	5.31 a/30	23.34 b/14	716.59 b/30	217.08 b
		30	14.22 a/6	3.1 a/10	38.65 c/10	184.68 a/14	60.16 a
3	*A. albertensis* UAMH 2976	18	103.85 b/6	45.06 a/30	32.13 b/6	71.95 a/14	63.25 b
		22	101.34 b/6	3 a/30	46.72 b/10	106.93 a/21	64.5 b
		25	30.43 a/14	4.12 a/21	14.27 a/21	79.69 a/14	32.13 a
		30	86.16 b/6	3.89 a/10	70.04 c/6	197.45 b/21	89.38 c
4	*A. carbonarius* Austria/grapes	18	19.9 b/6	12.32 bc/6	6.29 b/6	6.81 a/6	11.33 c
		22	2.93 a/6	8.63 bc/6	3.98 b/6	4.17 a/21	4.93 b
		25	6.51 a/6	3.37 ab/6	4.65 b/6	4.06 a/6	4.65 ab
		30	1.83 a/6	<LOD a/6	<LOD a/30	8.14 a/30	2.5 a
5	*A. carbonarius* CBS 127.49	18	<LOD a/30	<LOD a/30	<LOD a/30	2.9 a/30	0.73 a
		22	<LOD a/30	<LOD a/30	<LOD a/30	0.89 a/21	0.23 a
		25	<LOD a/30	<LOD a/30	0.5 ab/21	<LOD a/30	0.13 a
		30	<LOD a/30	<LOD a/30	0.74 b/21	1.24 a/21	0.5 a
6	*A. carbonarius* Armenia	18	4.45 a/10	3.14 a/6	2.25 a/10	4.66 a/14	3.62 a
		22	7.67 ab/21	3.07 a/6	2.77 a/10	5.12 a/14	4.65 a
		25	9.28 b/21	<LOD a/10	<LOD a/30	3.27 a/21	3.14 a
		30	4.26 a/21	0.6 a/30	3.22 a/14	7.86 a/14	3.99 a
7	*A. sulphureus* CBS 550.65	18	218.38 b/21	32.45 b/14	495.96 b/21	461.08 b/21	301.97 c
		22	246.79 b/21	63.37 c/14	518.93 bc/21	545.86 c/21	343.74 d
		25	40.74 a/10	17.62 a/14	551.94 c/21	79.68 a/21	172.49 b
		30	24.42 a/21	20.94 a/21	11.07 a/30	20.11 a/21	19.13 a
8	*A. westerdijkiae* Slovakia KMi9	18	28.81 b/10	32.45 b/30	124.52 c/6	479.74 d/10	158.27 c
		22	25.74 b/21	6.94 b/30	14.64 a/10	144.94 b/10	48.06 a
		25	88 c/10	12.96 c/10	311.58 d/10	33.85 a/14	111.6 b
		30	1.79 a/10	2.68 ab/6	56.5 b/10	395.61 c/10	114.14 b
9	*A. westerdijkiae* Slovakia KMi12	18	79.83 c/6	9.44 b/21	24.81 a/14	302.98 ab/10	104.26 a
		22	57.69 b/10	15.64 b/21	20.73 a/10	372.03 b/10	116.52 a
		25	36 a/10	16.2 b/10	137.26 c/10	591.28 c/10	195.18 b
		30	64.57 bc/10	2.88 a/10	101.36 b/10	255.29 a/6	106.03 a

Notes: CYA – Czapek yeast agar, MEA – malt extract agar, PDA – potato dextrose agar, YES – yeast extract sucrose agar, t – cultivation temperature, averages followed by the same letter (in columns for each strain) are not statistically significantly different on 0.05 significance level according Tukey test
